# Development of a psychobiological nosology for fear and frustration in clinical animal behavior using dogs as a model species

**DOI:** 10.3389/fpsyg.2026.1888439

**Published:** 2026-09-22

**Authors:** Beverley M. Wilson, Carl D. Soulsbury, Daniel S. Mills

**Affiliations:** School of Natural Sciences, University of Lincoln, Lincoln, United Kingdom

**Keywords:** affect, clinical animal behavior, dogs, fear, frustration

## Abstract

Assessing emotion is an integral part of the diagnostic process in clinical animal behavior and facilitates more specific management for the related behavior. Fear and frustration are commonly involved in such issues, and clinical animal behaviorists appear to find these emotions challenging to identify and differentiate, with a range of views on the nature of these emotions. This problem is compounded by a lack of consensus definitions for these terms, authors frequently relying on intuitive understanding, and the variability in definitions used, when they are actually defined, within the clinical animal behavior literature. We therefore sought to critically appraise existing clinical animal behavior literature on fear and frustration, before proposing a more formal functional psychological and biological (psychobiological) framework, which was inspired by work describing the evolved function of disgust. Our proposed definitions for fear and frustration structure and unite existing theories across the biological and psychological sciences with prominent research in the clinical animal behavior field, to provide a consistent frame of reference (nosology) for future research and clinical practice. We have used dogs as a model species to highlight how existing clinical literature can be usefully aligned into evolutionary-relevant themes relating to ecological context and key cognitive capacities. The outcome has the potential to not only be practically useful, in helping to standardize the dialogue relating to these emotional states, but also the methodology used could be more widely applicable to help improve future scientific research on affect.

## 1 Introduction

Clinical animal behavior (and veterinary behavioral medicine) is a comparatively new area of specialism within both the applied behavior and veterinary fields (Tuber et al., 1974; Mills and Ewbank, 2016). As with any new area of scientific endeavor, pioneers have proposed different approaches, with the main three recognized approaches in this field being one based on medical models of abnormal behavior; one based on the application of the learning traditions and principles of behaviorism and thirdly, the psychobiological approach which is grounded in contemporary affective neuroscience (Mills and Ewbank, 2016; Mills, 2022). These different approaches ultimately have the same goal of providing a framework for understanding problem behavior but take fundamentally different ontological perspectives on this issue.

The “medical” approach tends to consider problem behavior in terms of brain pathologies which need to be diagnosed and often physically treated, frequently using medications to correct what are seen as molecular abnormalities (Overall, 1997; Pageat, 1998). In contrast, the “behaviorist” tradition focuses on applying established reinforcement principles to understanding how a problem has developed, such as that used within human applied behavior analysis (Friedman, 2007). The “psychobiological” approach seeks to place problem behaviors within the context of animals following evolved behavioral rules and tendencies related to underlying psychological processes (Mills, 2023, 2025a). In the absence of reliable biological markers of specific mental states or disorders, reference to internal constructs such as “motivation” and “emotion” are considered essential if we are to avoid simply using behavioral syndromes as the basis of a diagnosis.

Although the use of behavioral syndromes may be superficially attractive, as they may have similar, objectively observable characteristics, they are inherently problematic as they may mask psychological heterogeneity (Mammì et al., 2026). As a result, interventions based on a syndrome used diagnostically will necessarily be non-specific or incomplete (Mills, 2003, 2022). Syndromes may also be superficial and covertly subjective in terms of the level of behavioral complexity they consider. For example, closer inspection of the broad behavioral syndrome often referred to as separation anxiety in dogs reveals four distinct types of behavioral reaction to being left alone, which appear to be based on different psychobiological responses to the same event (de Assis et al., 2020).

Ultimately a clinical animal behavior diagnosis is an inference about the state of another and so it is essential to recognize and accept the importance of the inference of internal states, like specific forms of emotional arousal. Indeed, emotional state is given importance within all three of the main approaches to clinical animal behavior, however, each of these approaches integrate the construct of emotion quite differently. The medical approach focuses largely on emotional dysfunction (e.g., Overall, 1997), using language such as noise phobias to distinguish these responses from normal fear responses; whereas the behaviorist approach considers emotion in terms of how it is affected by learning and its role as a reinforcer (Moore, 2011). In comparison, the psychobiological approach sees normal emotional functioning as integral to the expression of much behavior, utilizing component process theory (Scherer, 1984) to evidence specific emotional states (Mills, 2017).

A recent study by Wilson et al. (2024) highlighted the challenging nature of the clinical definition (and thus recognition) of specific emotional states underpinning problem behavior by those working with animals. Inconsistencies in terminological definitions used by practitioners arise from the approach used, the degree to which sound logic is applied and potential subjectivity bias (Wilson et al., 2024). There is therefore a need to revisit the basic principles involved in the definition of the affective state of another. Regardless of the clinical approach adopted, one of three common methods of definition are used: operational, intensional or ostensive. All may be useful, but they also have potential limitations as illustrated below.

1. Operational definitions are used to specify how a phenomenon is to be measured and thus model the phenomenon of interest (Stevens, 1935). This approach can be useful as it provides the opportunity to explore superficially similar phenotypes, through examination of underlying structure (Ribes-Iñesta, 2003), but it is at risk of subjective bias when items refer to an internal state dependent on the perceptions of an observer. Nonetheless, this approach has been used to psychometrically define emotional constructs such as frustration as a trait in dogs (McPeake et al., 2019). The Canine Frustration Questionnaire consists of a series of attitudinal statements relating to a dog’s behavior in various circumstances, and despite the risk of subjective bias, it has shown good external validity through correlation with more objective physiological and operationally defined behavior in standardized tests (McPeake et al., 2021).
2. Intensional definitions specify the essential properties of what is being defined. Intensional definitions based purely on observable behavioral attributes, in effect, specify behavioral syndromes, which are subject to the concerns already discussed. Nonetheless, intensional definition, is widely adopted within medicine through the specification of the necessary and sufficient criteria that must be met for a state to be classified in a particular way. This approach has its proponents within the field of clinical animal behavior, for example Overall (1997). Thus, for example, she defines “Fearful behavior (*sic)*/Fear” accordingly: “Necessary and Sufficient: Behavior that occurs concomitant with behavioral and physiological signs of fear as identified by withdrawal, passive and avoidance behaviors associated with the sympathetic branch of the autonomic nervous system and in the absence of aggressive behavior”. However, it can be difficult with such an approach to determine when some of the truly objective criteria are occurring, such as “behaviors associated with the sympathetic branch of the autonomic nervous system”. Thus, what might appear more objective is also at significant risk of subjective bias. Indeed, in the example provided here, it is also difficult to be precise about what is meant by “passive” with any reliability, and without potentially slipping into a tautology.
3. Ostensive definitions rely on examples to illustrate the phenomenon (Kotarbinska, 1960), i.e., they extend the definition by reference to external examples (they may thus be considered a form of extensional definition in contrast to intensional definition). For example, Masson et al. (2024) group together categorical phobias, sensory deprivation syndrome and post-traumatic stress disorder under their definition of “Fear Axis Disorders” in the dog. While the use of examples often has an intuitive appeal and may be easy to relate to, it very often lacks objectivity and often the lack of a comprehensive list means the definition is incomplete, unless a “catch-all category” like “unspecific disorder” or “condition not otherwise specified” is also included. Indeed this approach, alongside intensional definition of the more specific conditions making up the set, has been used historically in texts like The Diagnostic and Statistical Manual of Mental Disorders in humans. This approach is increasingly being questioned in terms of its scientific and biological validity (e.g., McLaren, 2010).

Given the importance of the issue of problem behavior definition for diagnosis (nosology) and thus the management of behavior cases, it is perhaps surprising how little attention has been given to the fundamental principles underpinning the definition of underlying psychological states. As highlighted, by Wilson et al. (2024), there seems to be a blind-spot, allowing the acceptance of an individual personal definition, i.e., the construct means what the author wants it to mean, often without further specification. This poses significant issues for scientific communication and the clinical development of the field.

Elsewhere, others, e.g., Mobbs (2018); Tybur et al. (2013, 2018), have identified issues with the conceptual consistency of definition of emotional states more generally, and the problems this poses, unless the definition is well grounded within an evolutionary, and thus ecological, context. However, Tybur et al. (2013, 2018) provided a useful framework for addressing this problem, using the primary emotion of “disgust” as an exemplar. They aligned diverse views and theories relating to this emotion to create a functional definition which was consistent with both the initial fundamental evolutionary advantage it provided and has continued to provide across its phylogenesis within human cognitive evolution. They demonstrated how this emotional response has maintained a similar biological function (rejection of something that might be harmful) over a wide range of ecological contexts, associated with varying levels of cognitive development. Such a framework maintains biological consistency largely through evolutionary exaptation. Thus, the initial evolution of disgust is associated with the advantages conferred through toxin avoidance, but this develops through natural selection to enable similar responses to stimuli that help facilitate pathogen avoidance and ultimately more psychologically complex constructs that might limit biological fitness, such as certain more indirect and morally-judged practices. It is proposed from this model that whilst the basic function is maintained, the specific context changes based on the psychological development of the individual. Thus, the rules governing the elicitors for the response change alongside related cognitive abilities, potentially resulting in a different experience. Such a framework provided an elegant method to intensionally define natural psychobiological boundaries to different forms of disgust. The approach maintained a biological coherence for different levels of emotion, as it respects both the ecological and cognitive diversity that may occur in emotional responses. It also provided a framework that is scientifically defensible for considering the emotional states that may exist in other species, that potentially avoids anthropomorphic bias.

Tybur’s approach is consistent with the evolutionary framing of psychological phenomena used within the psychobiological approach developed for clinical animal behavior (Mills, 2023, 2025a). This supposes that certain unconditional stimuli are used to create a stimulus set for some form of functionally-related emotional category, referred to as an emotional affordance category (Mills, 2025a,b). For example, looming/shadow projection, predator odor, unpredicted intense stimuli or changes in the environment, large discrepancies in expectation, etc. may all be grouped into an emotional affordance category relating to potential significant threats to survival; i.e., unconditioned fear-eliciting stimuli. The resulting emotional response is thus referred to as a fear response. However, there has not been wider ecological and cognitive consideration or framing of these emotionally competent stimuli in clinical animal behavior, in the way that Tybur et al. (2013, 2018) has done for disgust.

This paper proposes a functional psychobiological framework for defining emotions in non-human animals (using dogs as a model species), grounded in evolutionary function, ecologically relevant elicitors, and associated cognitive appraisal processes. By applying this approach to fear and frustration, we aim to provide a methodology for creating biologically coherent and clinically useful definitions that resolve existing inconsistencies in the literature. Accordingly, the definitions will be presented according to the following dimensions: (1) category (biological function); (2) emotionally competent stimuli; and relevant appraisals. Although inspired by the principles outlined by (Tybur et al., 2013), this framework is not intended to replicate their operational structure, but instead applies psychobiological principles to fear and frustration with the aim to provide clarification for the use of these terms.

We will initially review the clinical usage of the terms *fear* and *frustration* within a sample of the clinical animal behavior literature and critique the definitions used for these terms before we consider how these emotions can be conceptually considered. Finally, we will propose a clinical nosology to help address the problems identified with the existing usage of the terms.

## 2 Methodology

### 2.1 Review of the clinical usage of the terms fear and frustration

In light of the authors’ concerns with the existing terminology in the field, as detailed above, a targeted assessment of the clinical usage of the terms *fear* and *frustration* was undertaken at the start of this work (in 2022). The aim of this targeted literature search was to evidence if our concerns were present within the wider field. As this was a targeted review, an electronic search was conducted using the Web of Science online database to capture a sample of the published literature within the field. Dogs were used as the focal species on which this work is based, so the search terms ‘Dogs AND fear’ and ‘Dogs AND frustration’ were used. This produced a total of 465 papers relating to fear, dating from 1971, with the highest number of publications (49) per year occurring in 2021 (Supplementary Material 1). Forty-two papers relating to frustration were identified, dating from 1990, with the highest number of publications (6) per year occurring in 2020 (Supplementary Material 2).

The publications were screened, duplicates, those not written in English and those where the full-text was not accessible were excluded. Next, the remaining papers were screened for relevance ensuring the paper discussed fear and/or frustration. At this stage, we removed any paper which referred to fear of pain, as we viewed this as functionally distinct from fear in line with the psychobiological classification of emotion proposed by Mills (2025a,b). We consider fear of pain separately from fear as there are considered to be differing motivations and physiological responses involved in fear of pain (Bolles and Fanselow, 1980). For the purpose of this work, we wanted to consider fear only as it relates to avoidance of harm, not in relation to healing and protection of injuries, which we consider relates to the PAIN system (Panksepp, 1982, 1998).

Furthermore, the remaining potentially eligible papers were assessed for their relevance to fear and frustration (Figure 1) by screening titles and abstracts. Papers were excluded if they were not deemed relevant, for example, if the topic was human fear of dogs. Additionally, those whose focus was ambiguous or poorly defined were excluded when this became apparent, as we could not clearly determine if the papers were referring to fear or frustration. As such, we excluded publications which focused on “anxiety” [as this term can be used in relation to both fear and frustration, see Mills (2025a)], “stress” (as this broad term can be used to refer to many types of responses ranging from physiological exertion of indeterminate valence through to negative affective states) and “reactivity” (as this term can be used in many ways, such as descriptively to detail that an animal responded to a trigger without considering the inferred emotional response).

**FIGURE 1:**
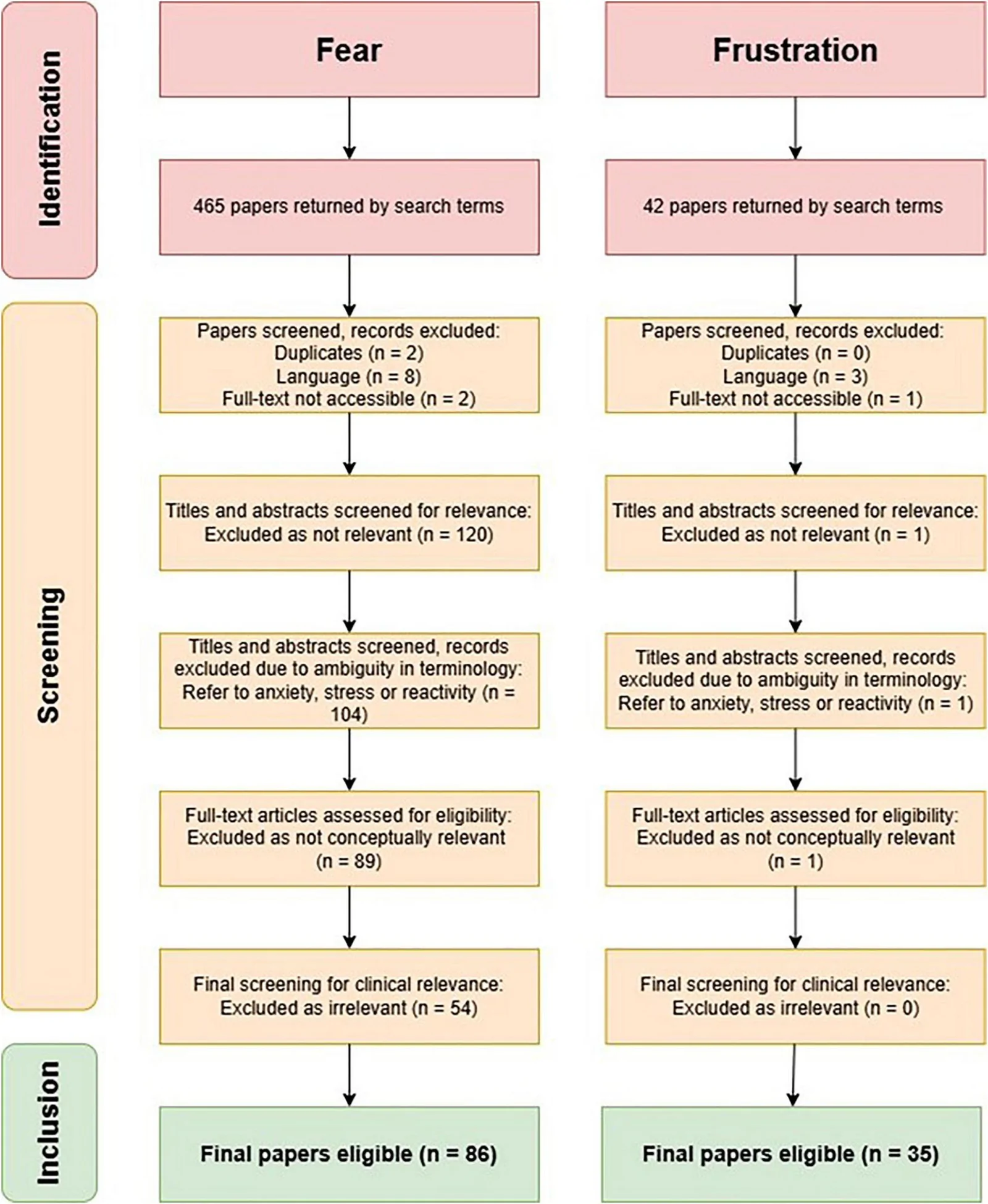
A flow diagram showing the approach to screening the publications identified by the search terms for a targeted review of the clinical usage of terms. The diagram illustrates the exclusion criteria and total number of papers considered eligible for inclusion in the review of clinical usage of terms within a sample of the literature.

The remaining papers were read in full and further screened to ensure they were conceptually relevant in the sense that they considered the emotions of fear and/or frustration in dogs. Irrelevant papers were excluded, for example, papers discussing the age of onset of a problem behavior were excluded. A final screening involved ensuring the remaining papers were of clinical relevance (that is, they had clinical utility). The process is summarized in Figure 1 (below).

For this screening process, all the papers were screened by the first author (BW) and reviewed by the last author (DM), as both are clinical animal behaviorists. Any disagreements or uncertainties over papers were resolved by discussion.

### 2.2 Critique of the existing terminology

The final papers considered eligible (86 in relation to fear and 35 in relation to frustration) were examined again in detail and the full text assessed. If a definition was provided in the paper, the type of definition used – operational, intensional or ostensive, was assessed by BW (and summarized using Google NotebookLM, UK version for ease of presentation to the other authors) (Supplementary Materials 3 and 4) and reviewed by DM. The summary provided by Google Notebook was carefully scrutinized and compared with the original source. Any errors or misrepresentations were corrected by BW. Any uncertainties of the classification were discussed among authors and a consensus reached.

### 2.3 Conceptual analysis of fear and frustration

We (BW, CS, DM) examined and reflected carefully on the basic biological function underpinning the use of the terms “fear” and “frustration” across the breadth of the definitions used within the clinical literature, and discussed this in relation to the wider literature. We tried to elucidate unifying themes, common features and discriminating factors. This allowed us to propose “base definitions” based on the general function of the emotion. We distinguished between motivation and emotion, assuming the hierarchical relationship between them as described in the psychobiological approach of Mills (2025a).

We used these base definitions to identify specific ecologically-meaningful contexts and the related cognitive appraisals required for defining each emotion. To do this, we extracted pertinent information, including any specific triggers or contexts described for each emotion, and any biologically relevant explanations. We used a reflexive process (Braun and Clarke, 2006) to categorize each emotion further based on biologically meaningful variations in function (inspired by the work of Tybur et al. (2013). Thus, the base definitions of each emotional state were elaborated into biologically relevant variants.

It is important to note that a significant proportion of the existing clinical behavior literature on fear and frustration does not include overt definitions, and many texts use contextual labels for convenience. These labels may be descriptive (e.g., aggression), phenotypic (e.g., sound sensitivity), motivational or contextual (e.g., repulsion). We therefore reviewed this clinical literature cautiously and avoided making assumptions or inferences about the underlying emotion(s) beyond what was explicitly stated. For example, in many texts, territorial aggression appeared to be used as a motivational label for a presenting problem (Borchelt, 1983; Landsberg et al., 2003; Dyjak et al., 2021). When such labels were encountered, we examined the full text to determine whether the author(s) specified which, if any, emotional state(s) they were referring to and how they defined or conceptualized these emotions. If this could not be established with confidence, that aspect of the text was excluded from our consideration of related emotional states. However, the presenting problem itself (including the stimulus and its associated characteristics) was retained as part of the iterative process used to consider ecological contexts and cognitive abilities. In this context, “cognitive abilities” refers to the cognitive processes involved in perceiving, interpreting, and appraising the stimulus within its ecological context, rather than to general cognitive capacity.

Throughout the development of the functional definitions, an iterative process was undertaken in which the authors attempted to apply these definitions to hypothetical cases and scenarios to identify any errors or omissions in the proposed definitions. This allowed continuous refinement of the definitions during the process.

## 3 Results

After conducting the targeted review of the clinical usage of the terms *fear* and *frustration*, we examined both the psychobiological nature of fear and frustration and how these terms fit within the broader evolutionary, comparative, and affective-science literature. We structure the results for each emotion in a similar way; this necessarily involves an element of discussion and argument to justify the proposals we make as a result. The main discussion examines the implication of these findings more broadly.

### 3.1 Proposed clinical nosology

#### 3.1.1 Fear

Within the existing literature, fear is widely considered to occur in relation to direct threats of harm (Blackwell et al., 2008; Mobbs, 2018) and these threats can be subcategorised as coming from social or non-social sources (Serpell and Hsu, 2001; Svartberg, 2005; Hsu and Sun, 2010; McGreevy et al., 2013). Trait-level fear may be assessed psychometrically within the construct of negative affect (Sheppard and Mills, 2002), or through the collective profile of individual responses (e.g., Espinosa et al., 2025). When the phylogeny of fear is considered, it is commonly described as offering protection from risks of physical harm that impact survival (King et al., 2003; Hydbring-Sandberg et al., 2004), with potential selection against the sensitivity of the trait during domestication (Hare and Tomasello, 2005; Hare et al., 2005; Kaminski and Nitzschner, 2013), as humans act as a buffer to many personal threats to the animals that live with them (Hemmer, 1990; Range and Marshall-Pescini, 2022). These descriptions are consistent with the wider biological literature on fear as an emotion, which often considers it to have adaptive value by helping animals escape and avoid danger (Panksepp, 2011). This danger may come from several different sources. Thus, we propose the base definition of fear as:

*An emotional response which arises when an individual predicts a threat of physical harm (damage) to itself. The function of the fear response is survival of the individual in the face of risk.*

The first two levels of elaboration of fear, which are clearly identified within the literature (see above) are fear in relation to aspects of the physical environment (e.g., noise fears, Blackwell et al., 2013; Storengen and Lingaas, 2015) and fear in relation to the social environment (social fears and anxieties, Puurunen et al., 2020; Stellato et al., 2017). Distinguishing between fears at this level (and within certain types of social interaction as argued below) is in line with the widely recognized hypothesis that unpredictability, sociality and social complexity are important drivers of cognitive evolution (Byrne and Bates, 2007).

In the case of the risk of harm from the physical environment, e.g., from the sound of a firework (Mills, 2005), there can be expected to be specific stimulus characteristics of relevance (emotionally competent stimuli – ECS) that have the potential to trigger the response (King et al., 2003; Gähwiler et al., 2020). These might relate to physically definable features like the suddenness and/or intensity of the stimulus, (e.g., in the case of noises, unexpected loud sounds are a common issue in dogs (Fagundes et al., 2018)), and these physical properties may be unconditioned triggers for a fear response (Riemer, 2019; Bremhorst et al., 2024). The fear response to these ECS may be modified by experience; it may be attenuated using desensitization and counterconditioning protocols (e.g., (Levine et al., 2007; Levine and Mills, 2008; Riemer, 2020); but the set of stimuli eliciting such a response may also be extended through learned associations (Sherman and Mills, 2008). Thus, a dog startled by the noise of a firework at a bonfire party, may also come to show a similar fear response to other features of the physical environment (like a faint smell of smoke) by association (Grigg et al., 2021).

In the case of fears related to the social environment, the situation is more complex. In line with the recommendations of Gajdoš Kmecová et al. (2021), we note the value of reflecting carefully on how the term ‘social’ is used in relation to emotional responses, from a psychobiological perspective. It is clear that the nature of the harm coming from social stimuli is associated with two biologically and evolutionarily distinct types of interaction that vary in their behavioral complexity and predictability: contest and predation (Samuel et al., 2020). In a contest there are competitive interactions between the actors, and so there will be signaling by both parties that need to be responded to. This not only relates to factors associated with communicating the risk of harm of a prolonged encounter, like resource holding potential (e.g., display quality, vocalization pitch, etc.) and/or the willingness or not to use specific strategies to gain a resource, but also the desire to terminate the contest (Hardy and Briffa, 2013). Whilst a contest involves competition over a resource and so may have its roots in frustration related responses (see below); fear may be expressed by one or more party depending on the perceived risk involved in the encounter. This insight helps to explain the ambivalent signals reported in agonistic encounters in both dogs (Déaux et al., 2015) and wolves (Fatjó et al., 2007). However, if the risk is assessed as too great, then signs of fear may be evident, or perhaps ritualized signals with a phylogenetic origin in fear (Van Der Borg et al., 2015), without necessarily invoking the original affective state. In this case the fear response will be enveloped within the broader context of the social exchange and related emotions (see below). By contrast, when faced with the risk of predation, the complexity of interactions is clearly much less (Samuel et al., 2020). The goal of potential prey is simply one of avoidance leading to survival, rather than complex social interaction. Even though escape may, in some instances, depend on displaying signals of fitness and thus risk of harm from prey toward predator; these are generally reliable signals that do not require the complex cognitive abilities of social interactions. The complex, calibrated responses of intersubjective contests, such as those involved in deference or other social strategies aimed at appeasement which occur in social contests, obviously do not feature in predator-prey interaction. Thus, the emotional fear response related to predation is ecologically (and cognitively) distinct, being more specifically focused on the survival strategies of non-detection and escape once detected.

Thus, we propose three primary, biologically-relevant categories of fear that are both functional and of clinical relevance. These are described further in Table 1. The categories within the table are labeled as well-established in the field, a plausible extrapolation, or a hypothesis for future testing, based on our evaluation of the strength and consistency of the existing literature and conceptual arguments supporting each proposed category. This is designed to highlight to the reader the evidentiary status of each proposed category and the degree of confidence with which it can currently be considered in its application.

**TABLE 1:** Proposed biological (functional) classification of fear in domestic dogs, related unconditional emotionally competent stimulus characteristics and associated appraisal.

Category (biological function)	Unconditional emotionally competent stimuli	Relevant appraisals
1. Physical fears: (avoidance of compromised biological fitness while navigating the physical environment) **Well established category**	Substantial prediction error in physical environmental monitoring Lack of predictive capacity, e.g., high-level novelty	a) Reduced ability to predict potential threats in the environment b) Prediction of harm c) Inability to predict d) Absence of signals of escape
2. Predator fear: (avoidance of predation-risk) **Well established category**	Predator odor, Shadow projection, Movement characteristics (velocity, acceleration etc.) proximity, trajectory of animate objects	a) Presence of a predator b) Predicted presence of a predator c) Detection by a predator d) Prediction of predator approach/attack
3. Social fears: (avoidance of harm during encounters associated with access or retention of resources) **Plausible extrapolation**	Biological correlates of resource holding potential Species typical threat behaviors and signals, Substantial prediction error in social interactions	a) Prediction of potential social conflict b) Evaluation of risk of harm from another versus value of contested resource c) Prediction of loss of contest
*4. Phobias and panic attacks:* *(Dysfunctional)*	*Not applicable*	*a) Lack of resources to cope* *b) Risk of extreme harm*

Proposed Categories 1–3 are considered to be functional, proposed *Category 4 (in italics)* represents what we consider as dysfunctional fear described in the literature. Bold text represents whether we consider the category is well established in the field, or a plausible extrapolation, or a hypothesis for future testing.

##### 3.1.1.1 Dysfunctional presentations of fear

In addition to these functional emotional responses, we identified a fourth category of fear in the clinical literature, relating to what are often referred to as some form of dysfunctional fear response. This was commonly associated with terms like “phobia” and “panic” (e.g., (Overall et al., 2001; Crowell-Davis et al., 2003; Dreschel and Granger, 2005). We propose these responses are maladaptive in the sense that they interfere with an individual’s ability to adapt to the environment, but in the absence of distinct biological markers, they may simply represent extremes of functional trait fear (see Mills, 2003 for further discussion of the distinction between maladaptive and malfunctional within medical paradigms of problem behavior). Given the lack of evidence that these represent evolutionarily differentiated emotional systems, it could be argued that these could be grouped under the categories of fear already described in this section. Nonetheless, for the sake of comprehensive clinical coverage, we have included this dysfunctional category in Table 1, with the caveat that they should probably be considered within the ecological context of three aforementioned types of fear described (physical, predator and social). Since our focus is on functional emotional states, we do not consider dysfunctional fears further.

#### 3.1.2 Frustration

Frustration is generally considered to be a negative emotional state (Bentosela et al., 2008; Bremhorst et al., 2022; Pedretti et al., 2022). It is often described within the context of denial, reduction or delay in rewards or incentives (Bentosela et al., 2008, 2009; Bremhorst et al., 2022), inequitable distribution of rewards (Range et al., 2012; Brucks et al., 2017a,b), inability to access desired resources (Lenkei et al., 2018; Bremhorst et al., 2019), violation of expectations (McGowan et al., 2014; McPeake et al., 2019; Santos et al., 2020) and/or an inability to express highly motivated behavior (Irimajiri et al., 2006; Starling et al., 2020), such as can occur when there are environmental constraints (Hewson et al., 1999; Lund and Jørgensen, 1999), or motivational conflict (Hewson et al., 1998; Pedretti et al., 2022). Common to all these contexts is the inability of the individual to fully execute motivated behavior (Hewson et al., 1999; Kuhne, 2016; Zurlinden et al., 2022). As with fear, frustration has also been specifically defined psychometrically as a temperament trait (McPeake et al., 2019). Frustration tolerance has also been considered at the level of a personality factor (Turcsán et al., 2018). Frustration has adaptive value through helping an individual to succeed in the face of restraint or restriction (McPeake et al., 2019), or to aid in self-defense when in imminent danger (Panksepp, 2011). It has been proposed that the ability to execute goal-directed behavior is a pre-requisite of frustration (Tomasello, 2022), as frustration is the failure of this process (e.g., de Assis et al., 2020). Thus, we propose the base definition of frustration as:

*An emotional response arising when a motivated behavior cannot be executed by the individual. The function of the response is to increase control over resources/the environment.*

It is worth clarifying at this point, that the failure to execute the motivated behavior can arise from both physical and/or psychological barriers as well as unmet reward expectancies (Hewson et al., 1999; Kuhne, 2016; Santos et al., 2020; Zurlinden et al., 2022). Within the clinical animal behavior literature, many problem behaviors, which on the basis of the above would be considered to be related to frustration, are given diagnostic labels that do not mention frustration; for example, territorial aggression (Borchelt, 1983), dominance aggression (Cameron, 1997), separation anxiety (King et al., 2000), stranger and/or fear aggression (Flint et al., 2017; Stellato et al., 2021) and compulsive disorders (Frank, 2013).

As with fear, we argue that there are ecologically important types of frustration associated with differing levels of cognitive complexity. Perhaps the cognitively simplest way, in which the execution of motivated behavior can be prevented is through the physical restraint of an individual. Thus, touch or predictors of touch and restraint may trigger a frustration response. Accordingly, resistance to touch or the anticipation of contact should be considered potential manifestations of frustration, since they relate to the predicted potential thwarting of activity and reduced autonomy (Jones, 2024). It is worth noting at this point that within the clinical literature, touch is generally described as a trigger of either a fear (Hsu and Sun, 2010) or pain response (De Keuster et al., 2006; Kuhne et al., 2012). Whilst not denying that these possible emotional responses may occur in relation to touch, there is clearly a need to appreciate the important theoretical argument, that responses like aggression in response to touch, or the anticipation of touch (such as reaching toward a dog), may be the result of a perceived potential limitation of autonomy and thus might be related to frustration in some situations. This may also explain why many dogs may resist touching of the feet, in particular (De Keuster et al., 2006; Kuhne et al., 2012), since this may be associated with a perceived loss of control of locomotion and thus control over the environment.

The skin and body surfaces may be considered particularly significant personal boundaries which trigger frustration associated with the potential inability to execute motivated behavior. However, the brain constructs many forms of functionally distinct maps of the space around the body (Previc, 1998) and invasion of these may result in similar psycho-behavioral responses, like aggressive threats. Peripersonal space describes the space immediately around the body. In primates this corresponds to the area in which objects can be manipulated, and beyond which exploratory eye movement typically occurs (di Pellegrino and Làdavas, 2015). In dogs, it has been suggested that there may be an equivalent to this which extends around 2m from the body, as their response to people varies within versus outside this distance (Fukuzawa et al., 2005). Anecdotally, our observations of clinical behavior cases note many instances of an attack triggered by invasion of this peripersonal space. Thus, we suggest that invasion of peripersonal space is another trigger of frustration that involves a form of ecological-enactive cognition (Rietveld et al., 2018). This describes how the individual constructs their world view and associated cognitive processes as a result of their interaction with the wider environment to create functionally meaningful perceptions (de Carvalho and Rolla, 2020). In the current example, peripersonal spatial boundaries are of particular significance and are thus psychologically represented as specific features that can affect their behavioral tendencies. Coello and Cartaud (2021) argue that peripersonal space is a critical zone of mediation between the body and the environment that is not only associated with control of goal-directed actions but also the organization of social interactions and relationships. Individuals maintain a preferred interpersonal distance, and violations of this boundary evoke discomfort and an urge to increase distance from the intruder (Welsch et al., 2019). Kennedy et al. (2009) highlight a potential role of the amygdala in the emotional response to invasion of peripersonal space, while Holt et al. (2014) demonstrate that the dorsal intraparietal sulcus and the ventral premotor cortex appear to preferentially respond to invasion of individual “comfort zones” related to interpersonal space by approaching animated stimuli. Vieira et al. (2020) further reported that a differential response in related regions associated with the invasion of this space by conspecifics as opposed to potentially threatening animate objects (invertebrates species who commonly evoke a fear response). This indicates the response is simply not related to fear, but rather protection of the peripersonal space. These findings and arguments suggest that the emotional response associated with invasion of peripersonal space is consistent with what we define as frustration rather than fear.

A further potential spatial boundary of similar behavioral significance, in that it can result in aggressive responses, relates to the space which contains defendable key resources. Perhaps the most commonly considered space is an individual’s territory (Lott, 1991). However, the behavioral flexibility required to respond to such circumstances is perhaps greater than the previous two contexts, which are very much more “close encounter” interactions. Territorial defense may involve protection of a whole territory (Smith and Parker, 1976) or specific resources within it (Schoener, 1987). Although defense takes many forms and involves the complexities of social interaction, there are clearly discernible, qualitatively different types of territorial contest (Hinsch and Komdeur, 2017). Humans tend to relate the limits of territories to features in the physical environment (geocentric features), but both cats (Fiset and Doré, 1996) and dogs (Fiset et al., 2000) may relate to such space more egocentrically (i.e., base them on the relative space around the individual). This would suggest that the perception of the territorial boundary may be another construct arising from ecological-enactive cognition (Rietveld et al., 2018) relating to the definition of the personal boundary to be protected. Thus, we argue that problems such as territorial aggression (Blackshaw, 1991; Beaver, 1994) may have their emotional roots in frustration related to invasion of this important personal boundary and the threat posed to control over (and thus motivated action toward) the related resources. This is in contrast to the emphasis given to fear in relation to territorial behavior in some popular general texts (e.g., Landsberg et al., 2023). Again, as with touch or invasion of peripersonal space, this does not deny the potential involvement of fear in interactions in this context. For example, if there is conflict at the boundary of a territory, the defender may show signs of fear in response to the threat of another (see previous section of results above). However, in this case the fear response relates to the nature of the interaction which ensues from territorial behavior, rather than being the primary emotional basis of territorial behavior *per se*. In support of this argument for territorial behavior being clinically related to frustration more than fear, it has been found that territorial aggression in Argentine Dogos occurs independently of aggressivity toward novel social stimuli, which might be considered more fear-related (Diverio et al., 2008). By way of further theoretical support for the proposal that territorial defense reflects a response with emotional roots in frustration, is the argument that this behavior not only represents the protection of a boundary but also the resources contained within it (i.e., a response to loss of potential sources of positive reinforcement) – a widely recognized model of frustration within the psychological literature (Amsel, 1992), discussed further below. It might also be argued that the psychological processes involved in the ecological-enactive cognition that facilitate the delimitation of a territorial boundary may also extend to define a similar boundary around other “owned” resources, resulting in possessiveness. We should highlight at this point that threats to these resources result in a particular type of social interaction, i.e., contest-related behavior, but this does not necessarily involve aggressive behavior. The strategy followed will vary with the perceived value of the resource being defended and predicted costs involved in acquiring or protecting the resource (Hurd, 2006). Value depends not only on need but also the investment made in the resources. Value is not fixed but varies over time. For example, in the case of an essential resource like food, value may depend on the how hungry the individual is, the availability of the resource, and in the case of certain resources like prey or a home, the effort made in acquiring it. When the resource is other individuals, such as offspring, siblings or reproductive mates, factors like individual biological investment e.g., presumed relatedness in the case of offspring or other family members, are important influences on perceived value. Perhaps, less widely considered, but equally important in shaping the strategy (including willingness) to defend a resource, is the nature of the relationship between those who own the resource and those seeking to acquire it. For example, from the perspective of the owner (who is the one generating a frustration response), certain resources may be given freely to others if they are part of their social group, if the immediate need of the resource holder is low. Where the threat to the resources is associated with a threat of harm, then the emotional response can be predicted to involve a blend of fear (personal threat to the defender) alongside frustration (threat to the resource). However, we emphasize again that not all threats to resources necessarily involve personal threats to the individual who owns them. Nor does a threat by an outsider need to be expressed as some form of aggressive behavior; rather any intrusion that is perceived by the defender to potentially thwart ownership of resources, can be considered a source of emotional frustration. Thus, perceived threats arising from the invasion of the space around an owned resource represent a distinct form of frustration, that encourages resource defense and protection behavioral tendencies.

Clearly there are also other potential threats to autonomy, which are not focused on the crossing of the spatial boundaries around owned resources, but are related to more general barriers to operating with an expected level of agency in the environment (Tomasello, 2022). This may include access to important, but unowned, or even shared, resources within a wider home range (Powell and Mitchell, 2012) or the violation of the general rules and expectations of a social interaction. Predictability may be greatly reduced in such situations and expectations may depend on a variety of factors including the current social situation. This may range from a dyadic interaction between two individuals through to more complex inter and intra group-level interactions. The ability to successfully navigate such social complexities and manage the related frustrations arising from unpredicted or uncertain social acts, may be a powerful evolutionary force shaping animal cognition (Tomasello, 2022). Thus, frustration within the broader context of social interaction represents another ecologically and cognitively significant phenomenon. As with responses around owned resources, the nature of the relationship between those involved in a social interaction may be important with regard to behavioral expectations and thus the frustration experienced. Clearly, differentiation must be made between individuals who are dependents (e.g., offspring) versus those that are potential mates or affiliates (siblings and other group members) who may all be part of an individual’s social group. It should also be noted that barriers to access or freedom of movement do not need to be absolute; it is possible that the loss may be partial. For example, an individual may be allowed to take some, but not all, of the food available, without causing frustration for others. This brings us to another form of frustration, i.e., frustration related to unmet reward-related experiences.

Within a learning context, frustration is often discussed in relation to the absence, reduction or delay in a predictable non-social reward (Amsel, 1992) and this has also been explored in dogs (e.g., Bentosela et al., 2008; Bremhorst et al., 2019; Kuhne, 2016). These studies indicate increased vigor in the previously reinforced response following frustration, as well as changes in facial expression, such as increased blinking, lip parting, jaw dropping, nose licking and flattening of the ears, along with a lateralised left shift in visual orientation, indicative of a negative emotional valence (Simon et al., 2025). From a behavioral motivation perspective, this experimental paradigm can be reframed in terms of the inability to execute a behavior with the goal of attaining a larger reward. Thus, unmet expectations constitute another important group of sources of frustration.

Finally, we identified another category of frustration-related responses associated with motivational conflict. These are classified as approach-approach, approach-avoidance and avoidance-avoidance conflict in line with the principles outlined in Lewin’s, 1935 field theory (Lewin, 2013). These states fit the requirements for the definition of frustration proposed here, insomuch as they reflect an inability to execute motivated behavior, if only because of competing motivations. They also meet the functional specification of reduced autonomy over the environment. Obviously, where the conflict involves avoidance, it is likely fear will also feature as part of the emotional response, but in the absence of a behavioral decision, we suggest the animal is fundamentally in a frustrated state.

Thus, we propose six primary, biologically-relevant categories of frustration that are both functional and of clinical relevance. These are described further in Table 2. Again, the same categorical labels of well-established in the field, a plausible extrapolation, or a hypothesis for future testing have been applied in the same manner as for Table 1.

**TABLE 2:** Proposed biological (functional) classification of frustration in domestic dogs, related unconditional emotionally competent stimulus characteristics and associated appraisal.

Category (biological function)	Unconditional emotionally competent stimuli	Relevant appraisals
1. Physical restraint (motivated to maintain autonomy and agency) **Plausible extrapolation category**	Restrictions to self, Restraint	a) Stimuli that cause restraint b) Stimuli that restrict an individual, or predict restriction
2. Invasion of peripersonal space (motivated to defend self and personal space) **Well established category**	Touch, Invasion of personal space, Sensory irritation	a) Touch, or anticipation of touch b) Invasion of another into personal space c) Sensory irritation d) Lack of access to safety
3. Invasion of space around (essential) defendable resources (motivated to protect resources) **Plausible extrapolation category**	Invasion of key spatial/territorial boundaries, Barriers to essential resources (including safety), Thwarted approach in the environment or via social interaction Protection of resources	a) An incursion by another into key spatial/territorial boundary b) Inability to access an essential resource c) Thwarting of access to safe space d) A threat to a resource already acquired e) Threat to a resource the individual is wanting to acquire
4. Social interactions (enabling development of and benefit from social structure) **Hypothesis for future testing category**	Species typical communicative signals, Substantial prediction error in social interactions Thwarting of breeding opportunities	a) Failure of another to conform behaviorally as expected b) Restrictions to autonomy within an interaction c) Lack of social cohesion d) Lack of reciprocation e) Lack or denial of reproductive opportunity f) Courtship failure g) Competition failure
5. Motivational conflict (motivated to resolve internal conflict) **Well established category**	Activation of two incompatible motivational systems leading to conflict in behavioral tendencies	a) Approach-approach conflict b) Approach-avoidance conflict c) Avoidance-avoidance conflict
6. Absent, reduced or delay in non-social predicted rewards (unmet value expectations) **Plausible extrapolation category**	Unmet expectation in reward contingencies, Reward prediction error Rewards lower than predicted value	a) Inability to access desired non-essential resource b) Threat to a resource already acquired c) Delayed access to incentives d) Access to resources of lower than predicted value
*7. Rage syndrome and stereotypies (Dysfunctional)*	*Not applicable*	a) Lack of resources to cope b) Persistent frustration

Proposed Categories 1–6 are functional, proposed *Category 7 (in italics)* represents what we consider as dysfunctional frustration described in the literature. Bold text represents whether we consider the category is well established in the field, or a plausible extrapolation, or a hypothesis for future testing.

##### 3.1.2.1 Dysfunctional presentations of frustration

As with fear, we also identified a further group of what are referred to as dysfunctional frustration responses. This was commonly associated with conditions like stereotypic behavior (Mason et al., 2007; Latham and Mason, 2010) and obsessive-compulsive disorders (Frank, 2013), but also certain manifestations of aggressive behavior (Cameron, 1997; Flint et al., 2017; Stellato et al., 2021). Again, for the sake of comprehensive clinical coverage, we have included this category in Table 2, but again with the caveat that they should probably be considered within the ecological context of six aforementioned types of frustration. Accordingly, we do not consider dysfunctional forms of frustration further.

## 4 Discussion

Despite having been discussed within the clinical literature for some time, it is clear that the constructs of fear and frustration lack consensus in this literature and their definition is often left to the reader’s opinion (Wilson et al., 2024). This is particularly apparent in relation to the longer history of writing about fear. Our examination of the linguistic use of the terms *fear* and *frustration* within this literature identified important inconsistencies, particularly where the related motivation or context described by the author(s) did not align with the presumed biological goal of the stated emotion (Cameron, 1997; Landsberg et al., 2023). In this regard, resource guarding (and associated terminology) provides a useful illustration of this issue. The threat of losing a resource is often described as a *fear*; however, this reflects a more colloquial use of the term, which perhaps better means an anticipation of something unpleasant. From a biological perspective the goal of fear-related responses is concerned with survival (including the avoidance of physical harm) (Panksepp, 2011). Accordingly, we argue that the term *fear* appears inappropriate when discussing the loss of a resource. In this circumstance, the threat is to control over a resource, not to the individual’s physical safety (McPeake et al., 2019). We propose that the threat of resource loss more closely aligns to psychological models of frustration (e.g., Amsel, 1992). A further example of the confusion around current linguistic usage relates to the term *anxiety*, which could plausibly refer to either fear or frustration, as discussed by Mills (2025a). This highlights pervasive effects of colloquial terminology on scientific rigor, which has practical ramifications that extend beyond the theoretical, for example when applied to clinical and welfare contexts.

Given the difficulties associated with the use of terminology within the clinical animal behavior field, we propose that, alongside the definitions presented here, terms with potential for ambiguity are used in a manner that clarifies the specific emotion to which the author is referring. For example, if the term anxiety is applied to a dog experiencing motivational conflict, we suggest including a clarifying statement such as anxiety arising due to frustration from unresolved motivational conflict. We consider this approach advantageous for the terms we considered as ambiguous within the literature, including anxiety, stress, and reactivity. From a clinical perspective, we hypothesize that a dog would utilize different coping strategies (including different behavioral tendencies and communicative signals in line with component process theory (Scherer, 1984) when responding to threats of harm to themself versus threats to a resource. This distinction, if explored further and found to be true, has the potential to influence clinical intervention strategies. For example, fearful dogs who are hypervigilant to environmental dangers may require interventions focused on reducing their response to mild threats which result in unnecessary vigilance (thereby reducing their fear). By contrast, dogs who are disturbed by loss of autonomy or loss of a resource will require interventions aimed at developing coping skills and resilience when control over their environment is reduced (Mills et al., 2013).

The linguistic inconsistencies no doubt contribute to the practical difficulties reported in clearly differentiating fear and frustration by those working within the clinical animal behavior field, which have been reported previously (Wilson et al., 2024). This is one of the factors that led us to review the clinical literature and propose definitions that are grounded in established biological and psychological theory. Each emotion has been considered in relation to its proposed biological goal, emotionally competent stimuli (Damasio, 2004) and associated behavioral tendencies (Scherer, 1984). Fear enables survival (Panksepp, 2011; Mobbs, 2018), with unconditioned threats arising from evolved cues predictive of physical harm such as environmental dangers, predators, or social attack. These threats often relate to a clear external stimulus, leading the animal to respond directly to the perceived danger. By contrast, frustration relates to autonomy and the primary biological function of resource acquisition (Hurd, 2006). Stimuli related to frustration may relate to external factors that thwart behavioral execution (such as restraint) or internal factors, including predictions relating to unmet expectations or centrally generated states involving motivational conflict, which limit agency (Tomasello, 2022). Regardless of the source, thwarting results in reduced control over the environment and potentially the experience of a loss of agency. Thus, although the general emotional outcomes of fear and frustration may appear similar in terms of core affect (valence and arousal), we argue that the behavioral response to these emotional states is distinct, justifying their separation along the lines presented (see Supplementary Material 5 for illustrated examples) (Mills et al., 2013; Mills, 2023, 2025a).

It is possible that what are described as pathological states in the clinical literature (Overall, 1997; Dreschel and Granger, 2005) represent instances in which animals perceive either an extreme threat in terms of fear or frustration or a combination of emotional states with synergistic effects on arousal. In either case, their secondary appraisal, in line with the theory of Frijda (1993), is then more likely to result in an evaluation that the challenge is either at the limits of, or potentially beyond, their coping ability (resulting in extreme arousal levels and more pronounced behavioral tendencies). In this situation, we hypothesize, intervention may be optimal if focused on altering their primary and secondary appraisal processes and coping mechanisms, including the social support available to them. This perspective of what are commonly termed “emotional disorders” or “dysfunctional responses” helps to explain a major challenge to the medicalisation of problem behavior: the general lack of clear biological markers of “pathological” affective states (Mills, 1997; Eronen, 2021). Reconsidering pathological states as potentially functional emotional states not only provides insights into potential alternative treatment options that align with the biological goals of the emotions involved, but it also potentially provides a more positive prognosis for these cases (as they are not seen as malfunctional) and understanding of their welfare implications.

Overall, we argue that the concepts of fear and frustration within clinical animal behavior require clearer theoretical grounding if they are to function as meaningful scientific and clinical constructs. By synthesizing findings from clinical animal behavior, psychology, affective neuroscience, ethology, and evolutionary theory, we have proposed a psychobiological framework that defines these emotions functionally according to their adaptive biological goals, ecologically relevant contexts, and associated cognitive appraisal processes. In doing so, we have provided an important bridge between component process theory of emotion and comparative psychology. This is not only of fundamental scientific value, but also clinical utility as we have sought to move the field of clinical animal behavior away from descriptive or syndrome-based approaches and toward biologically coherent nosology of affective states.

We used dogs as a model species as we were initially motivated by the practical challenges faced within clinical animal behavior, but also saw the opportunity to make a broader contribution to affective science and evolutionary psychiatry by addressing a longstanding conceptual problem: how emotional categories can be defined consistently across contexts, levels of cognition, and species while remaining grounded in evolutionary function. By aligning emotional states with the adaptive problems they evolved to solve, the approach offers a way to synthesize behavioral, motivational, and cognitive perspectives on emotion without reducing emotions either to purely subjective experiences or to superficial behavioral syndromes. By conceptualizing many apparently maladaptive emotional responses as potentially functional systems operating under conditions of potentially extreme appraisal, uncertainty, or reduced coping capacity, this perspective challenges strict pathological models of affective disturbance. This approach resonates with current developments in evolutionary psychiatry and dimensional models of psychopathology, where emotional states are increasingly viewed in relation to adaptive function and context sensitivity rather than categorical dysfunction.

Within this manuscript we focus primarily on functional, motivational, and appraisal-based aspects of emotion that can be assessed within a clinical psychobiological framework. We do not examine neural circuitry, physiological biomarkers, or developmental neurobiology, as these fall outside the scope of this article. Nevertheless, all elements of Tinbergen’s analysis of behavior are important to consider in relation to this work. Development is relevant to the interpretation of emotional categories; for example, emotions such as lust are unlikely to be evident prior to puberty, and conditioned triggers may emerge through classical conditioning with relevant unconditioned stimuli. We propose that considering these aspects further, and testing associated hypotheses, would be a useful direction for future studies building on this work.

Importantly, this framework also provides a comparative methodology for studying affect across species. The proposal that emotional systems retain functional continuity across phylogeny, while becoming elaborated through increasing cognitive complexity and ecological demands, aligns with contemporary evolutionary approaches to emotion and cognition. This enables a scientifically defensible approach to comparative affective science that avoids anthropomorphic projection, while also providing an alternative to the issues arising from behaviorism and more medicalised perspectives of emotional disorders. In this respect, the work contributes to ongoing debates concerning the nature of distinct emotional systems and psychopathology and the role of appraisal processes in non-human animals and the relationship between emotion, motivation, and behavioral control.

It is to be hoped that the work presented here may help to facilitate greater conceptual integration between animal and human affective science. Indeed, we suggest that the methodology developed here may be as important as the specific definitions proposed. The integration of evolutionary function, ecological context, emotionally competent stimuli, and appraisal processes provides a generalizable strategy for refining emotional constructs across affective science. Fear and frustration represent only initial exemplars of this approach. Future work may apply similar principles to other emotional states, thereby supporting the development of a more coherent and biologically grounded science of emotion across both human and non-human animals.

## Data Availability

The original contributions presented in the study are included in the article/Supplementary material, further inquiries can be directed to the corresponding author.
