# Supplementary material for: Development of a psychobiological nosology for fear and frustration in clinical animal behavior using dogs as a model species
Source: Front Psychol. 2026 Sep 22;17:1888439. doi: 10.3389/fpsyg.2026.1888439 (PMC13638404; doi:10.3389/fpsyg.2026.1888439)
Supplement: Supplementary file 1 [file Table_1.docx]

**Supplementary Materials 1**

A figure from the Web of Science core collection on the number of publications and citations relating to “Dogs AND fear”
