# Supplementary material for: Development of a psychobiological nosology for fear and frustration in clinical animal behavior using dogs as a model species
Source: Front Psychol. 2026 Sep 22;17:1888439. doi: 10.3389/fpsyg.2026.1888439 (PMC13638404; doi:10.3389/fpsyg.2026.1888439)
Supplement: Supplementary file 3 [file Table_5.docx]

**Supplementary Material 5- Illustrative examples of the proposed application of the definitions**

a) A threat to a resource, a potential elicitor of frustration
b) A physical fear and lack of access to safety, potentially eliciting both fear and frustration
c) An incursion by another into a key territorial boundary, a potential elicitor of frustration
d) An incursion by another into peripersonal space, a potential elicitor of frustration
